# *Agave* Chewing and Dental Wear: Evidence from Quids

**DOI:** 10.1371/journal.pone.0133710

**Published:** 2015-07-31

**Authors:** Emily E. Hammerl, Melissa A. Baier, Karl J. Reinhard

**Affiliations:** 1 Department of Anthropology and Forensic Science Program, University of Nebraska-Lincoln, Lincoln, Nebraska, United States of America; 2 Buffalo National River, National Parks Service, Harrison, Arkansas, United States of America; 3 School of Natural Resources, University of Nebraska-Lincoln, Lincoln, Nebraska, United States of America; NYIT College of Osteopathic Medicine, UNITED STATES

## Abstract

*Agave* quid chewing is examined as a potential contributing behavior to hunter-gatherer dental wear. It has previously been hypothesized that the contribution of *Agave* quid chewing to dental wear would be observed in communities wherever phytolith-rich desert succulents were part of subsistence. Previous analysis of coprolites from a prehistoric agricultural site, La Cueva de los Muertos Chiquitos in Durango, Mexico, showed that *Agave* was a consistent part of a diverse diet. Therefore, quids recovered at this site ought to be useful materials to test the hypothesis that dental wear was related to desert succulent consumption. The quids recovered from the site were found to be largely derived from chewing *Agave*. In this study, the quids were found to be especially rich in phytoliths, and analysis of dental casts made from impressions left in the quids revealed flat wear and dental attrition similar to that of *Agave*-reliant hunter-gatherers. Based on evidence obtained from the analysis of quids, taken in combination with results from previous studies, it is determined that *Agave* quid chewing was a likely contributing factor to dental wear in this population. As such, our method provides an additional avenue of dental research in areas where quids are present.

## Introduction

Quids are important but often ignored remains that can have importance in the interpretation of archaeological sites. In many cave sites in the United States and Mexico, quids are abundant in midden deposits. Many prehistoric peoples used high fiber, low soluble carbohydrate foods from *Yucca* leaf bases, *Agave* hearts, cactus pads, maize leaves, aquatic plant rhizomes, and other plants. Phytoliths, both silica and calcium oxalate, are present in these food sources. There has been disagreement over whether or not phytoliths are harder than tooth enamel [1–3], but recent work by Rabenold and Pearson [4] convincingly argues that phytoliths do indeed abrade enamel, although perhaps not in the way that is tested in previous work (most notably, in the studies by Lucas et al. and Sanson et al. cited above [1,2]). In particular, Rabenold and Pearson point out that there is significant evidence that repeated exposure to phytoliths can indeed result in the abrasion of dental enamel. This holds for some phytoliths that are softer than dental enamel and do not immediately result in the loss of enamel from the tooth surface upon initial exposure [4]. Since phytoliths can abrade tooth enamel in this manner, evidence of chewing quids made from such phytolith-rich plant foods can provide support for the assertion that specific foods are contributing causes of dental microwear, attrition, and tooth loss within specific populations [5].

**Fig 1:**
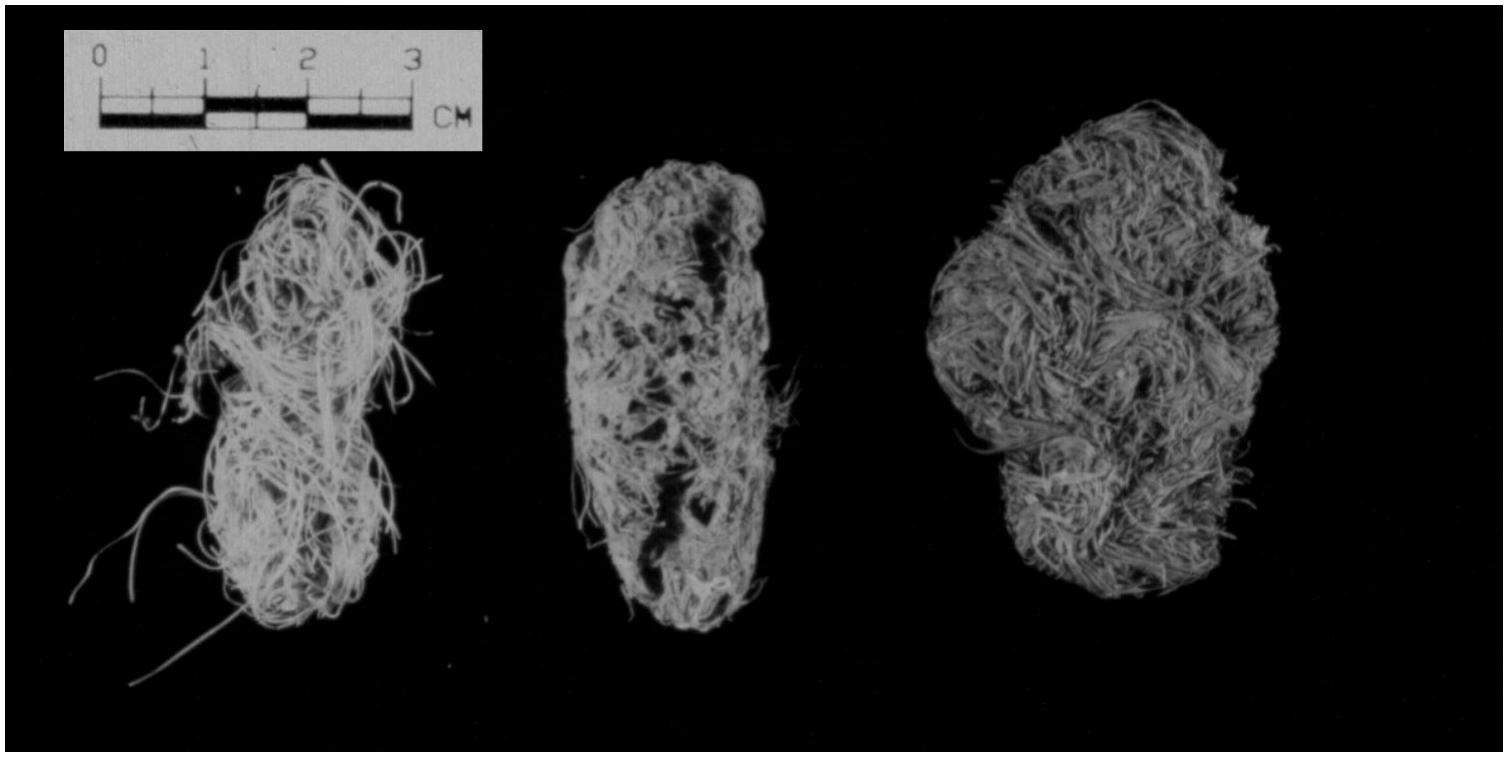
Example of an expectorated quid found in La Cueva de los Muertos Chiquitos, Durango Mexico. Based on the composition, it is easy to see how quid chewing (and swallowing) contributed to prehistoric high fiber diets and large amounts of phytoliths in the diet.

The anatomical makeup of *Agave* spp. plants is such that the majority of the fibers contained in these plants are indigestible. Therefore, when *Agave* is eaten, most of the plant is expectorated rather than swallowed, producing a large number of quids [6]. Only rarely do we find agave boluses in prehistoric coprolites. More commonly, we find massive accumulations of expectorated quids in dry caves (Fig 1).

**Fig 2:**
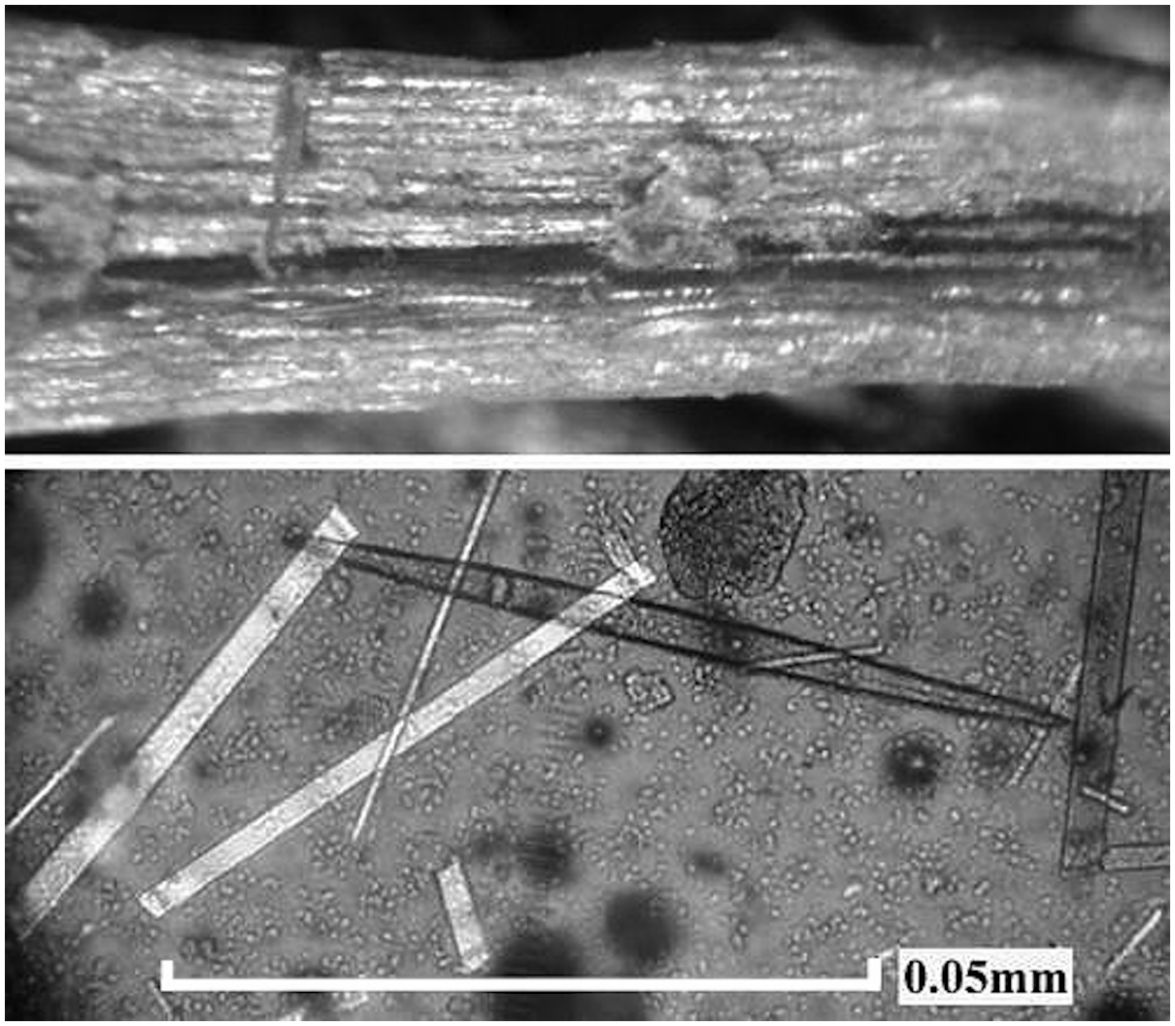
This shows phytoliths recovered from a quid. The upper picture shows a single fiber within an *Agave* quid. The parallel lines of reflective structures are rows of phytoliths. The lower picture shows raphid and rhomboid phytoliths recovered from an *Agave* quid. Although the percentage weight of phytoliths in quids is difficult to quantify, we observed phytoliths in every *Agave* quid.

In the case of *Agave*, the mastication of quids has serious implications for the condition and wear of the teeth. As shown by Danielson and Reinhard [5], phytoliths are present in high numbers in *Agave* and present with two types of calcium oxalate types: double-pointed, needle shaped raphids and chisel-shaped, rhomboidal crystals (Fig 2).

The physical examination of quids reveals impressions of teeth embedded in the fiber and dental casts from quid tooth impressions provide direct evidence of dental wear. Among other studies on quid analysis, Reed [7] analyzed tooth impressions left in quids found at Hoy House and Lion House in southwestern Colorado and Turner demonstrated that excessive dental wear and tooth loss could be seen in casts made from quids based the analysis of bite-marks on quids from a cave in Nevada [8].

Numerous studies in recent years have explored the relationship of phytoliths to dental wear in archaeological populations [1,9]. These studies have included modern experimental dental wear simulation [10] and direct analysis of phytoliths imbedded in the surfaces of archaeological teeth [11,12]. Other researchers have discovered phytoliths in dental calculus from archaeological contexts [13–15]. *Agave* and cactus phytoliths have been recovered from coprolites and have been implicated in dental wear [5,16]. These approaches, when applied to temporal variation in a single region, provide a basis for characterizing changes in dental wear related to diet [17]. Here we present an additional approach to the analysis of dental wear—casting prehistoric dentition from quids. This approach, when combined with botanical analysis of quids and coprolites from the same site, is a novel method for documenting dental wear and identifying potentially contributing dietary abrasives.

Quids are the expelled fiber masses that result from chewing vegetal matter [8]. In the current study, we conduct a detailed analysis of *Agave* quids found at La Cueva de los Muertos Chiquitos, Durango, Mexico, and have found that dental impressions are often visible in quids and can be cast. At this particular site, dental remains are only available for a total of seven subadult individuals that range in age from several months to five years. As such, it is an excellent example of the need for alternative methods of investigating dental growth and wear. Here, we propose that dental information can be obtained from examining dental casts made from quids. As the quids themselves can be analyzed for phytolith content when studied in conjunction with coprolite analysis, the prevalence of phytolith-rich foods in the diet can be assessed based on dental wear patterns.

Danielson and Reinhard [5] demonstrated that prehistoric dental microwear was caused by quid chewing among hunter-gatherers in the Texas-Coahuila borderlands. Danielson and Reinhard [5] explored dietary explanations for dental wear in the Lower Pecos Canyonlands. Previous research had shown that the Archaic hunter-gatherers in this region had remarkably high levels of severe dental wear [18–21]. These authors suggested that diet should be explored as a key factor in causing dental wear and abscesses in the region. Danielson and Reinhard [5] addressed the potential of calcium oxalate phytoliths as a cause of the wear. They tested the hardness of calcium oxalate phytoliths against dental enamel and found that the phytoliths were capable of scratching enamel. They then focused on agave phytolith arrangement within plant tissue, comparing this arrangement with parallel incised wear on ancient teeth. They found that the distance between parallel incisions on enamel surfaces was the same as the distance btween chisel-shaped phytoliths in plant tissue. Finally, they assessed the abundance of phytoliths and found that up to 20% of the solid volume of coprolite residues. These observations combined showed that calcium oxalate phytoliths were a consistent component of diet. Their abundance and hardness signals their strong potential as a source of parallel dental microwear as documented in the lower Pecos.

Furthermore, Danielson and Reinhard [5] hypothesized that similar patterns of dental wear would be seen in later, agricultural societies that continued to rely on desert succulents and so subsequently, these authors expanded their studies to other hunter-gatherer and agricultural peoples in the Southwest [9]. This research showed that reliance on desert succulent wild plants such as agave, yucca and prickly pear did not disappear with the advent of agriculture. *Agave*, for example, was cultivated by agricultural peoples in Mesoamerica and the Southwest [22–25]. Therefore, dental wear resulting from phytoliths was an aspect of these assemblages both before and after agriculture spread across the southwest.

In the current study, a detailed analysis of the quids was conducted to determine if quid chewing could be associated with dental wear as seen in casts made from tooth impressions in the quids. The primary aim of this study is to test Danielson and Reinhard’s [5] hypothesis which we approach in three ways: First, we seek to identify the botanical origin of the quids. Second, we document the phytolith content of the quids. Third, we test the feasibility of obtaining dental casts from the quids with sufficient resolution to show dental wear.

## Materials and Methods

**Fig 3:**
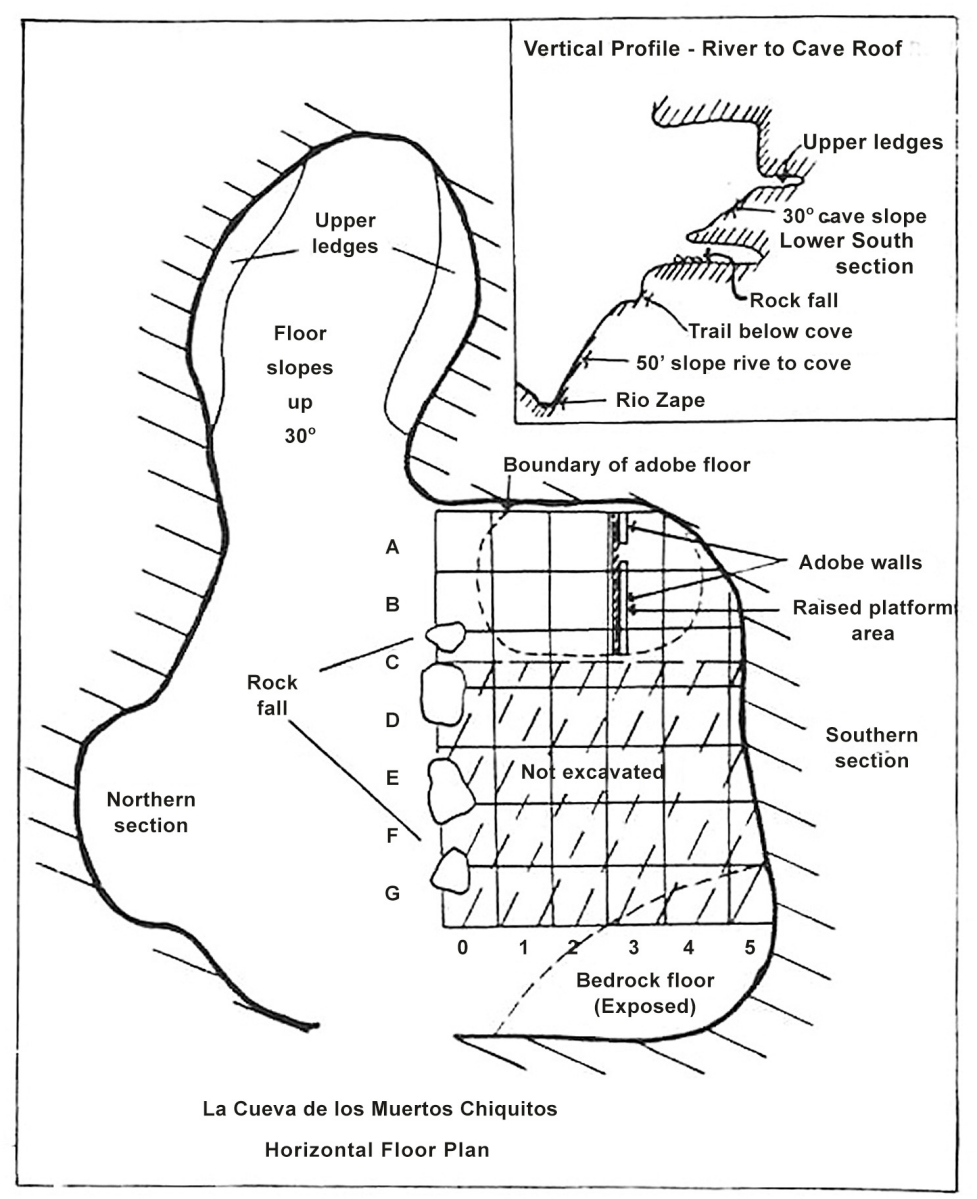
Map of La Cueva de los Muertos Chiquitos, Durango, Mexico. Map adapted from Brooks et al. [26].

La Cueva de los Muertos Chiquitos is located in the northwestern part of Durango, Mexico on the eastern slopes of the Sierra Madre Occidental and was excavated by Richard Brooks and colleagues in the early 1960s (Fig 3). The site lies at about 1800 meters in elevation and overlooks the Rio Zape. The cave is about 18.5 meters wide and 9 meters deep with a height ranging from 1.2 meters near the opening to half a meter near the rear [26,27]. The cave has been attributed to the Loma San Gabriel people, a little known but widely distributed culture [27]. The site is dated to 1200–1400 years ago by radiocarbon dating [26]. Physical remains from the cave included quids, coprolites, and human skeletons. From the collection of 2,890 quids that were recovered from a midden in the cave, 50 were selected for the present analysis due to the presence of clearly identifiable bitemarks. Specimens are catalogued as numbers Q 1–58 Cast Specimen Analysis ‘89 –Rio Zape Collections, and are held at the School of Natural Resources Palynology Laboratory at the University of Nebraska-Lincoln.

This study has three primary aims: First, we seek to identify the botanical origin of the quids. Second, we aim to document the phytolith content of the quids. Third, we test the feasibility of obtaining dental casts from the quids with sufficient resolution to show dental wear.

To determine botanical origin, we examined the quids with an Olympus SZ stereo zoom microscope. The fiber and epidermis morphology were examined to determine the botanical origin of the quids following guidelines of Bell and King [28]. Based on the observed morphology, the quids were then separated into *Agave* and non-*Agave*. Next, phytolith morphologies were examined to verify fiber and epidermis identification. A sample of 25 quids that had been visually identified as *Agave* and 8 quids identified as non-*Agave* were randomly selected from the total sample to be used in this process. The dry weight for each quid was recorded. Phytoliths were recovered from the quids following the methods for phytolith extraction described by Danielson and Reinhard [5] with minor modifications. The quids were placed in 600 ml beakers with 0.25 g potassium dichromate. An initial volume of 100 ml 50% hydrogen peroxide was then added and stirred. These mixtures were then briefly heated until bubbling reactions commenced. The reactions were quite violent and care had to be taken to prevent the solution from boiling over. To prevent spill over, 100% ETOH was carefully sprayed into the beakers to break surface tension. Goggles, protective clothing, and two pairs of latex gloves were worn to protect from chemical burns. Occasionally, the reaction was repeated in order to dissolve all of the cellulose in the fibers. When the fibers were successfully dissolved, the mixture was centrifuged and the supernatant was poured off. The extracted phytoliths were examined with a Jenaval APO DIC trinocular research differential interference contrast microscope at 450x and 1000x magnification. The extracted phytoliths were dried and weighed. The total phytolith content of the quids was determined by dividing the dry weight of the extracted phytoliths by the weight of the original quid.

**Fig 4:**
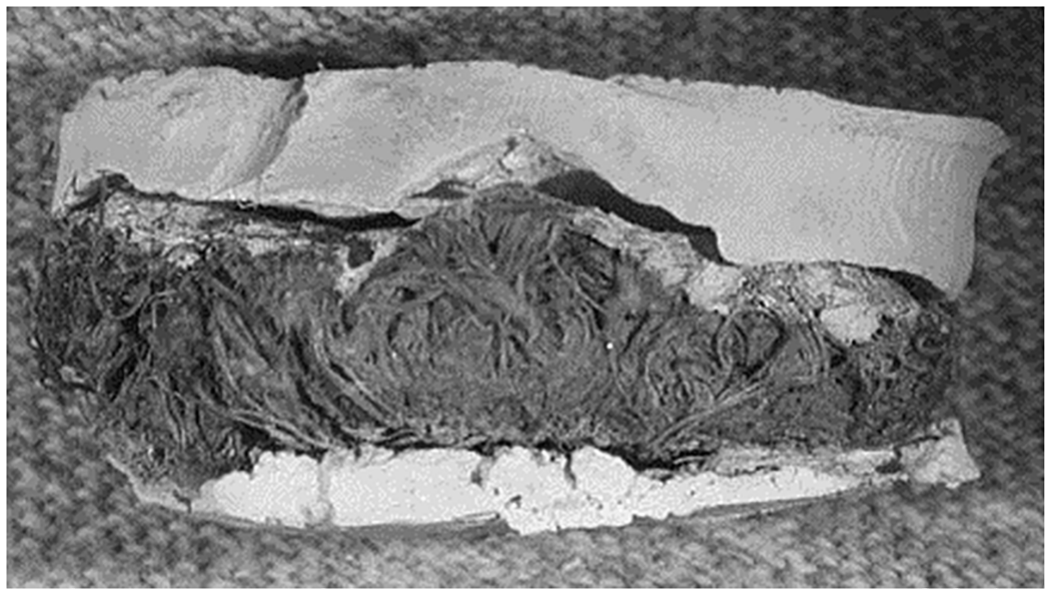
This picture shows Labstone casts separating from a quid after the dental wax was removed. In this case, partial casts of the upper and lower dentition were made.

Dental casts were made from quids with distinct dental impressions (n = 49). To make the cast, the quid was first coated with a thin layer of acrylic lacquer. This method was developed after trial and error attempts to make casts directly from the quid impressions. Without the acrylic lacquer, the casting material adhered to the fibers of the quid. We found that a very thin application of acrylic lacquer prevented breakage of the quid and did not obscure features of the cast. After this coating had dried, a ring of dental wax was made around the periphery of the impression in order to make a mold out of the quid. The casting material, made from a mixture of Labstone dental plaster and water, was then poured into the mold and allowed to dry^1^. After the plaster was set, the wax and cast were removed from the quid (Fig 4).

## Results

### Botanical origin

An overwhelming majority (80.4%) of the analyzed quids were derived from *Agave*. The remainder (19.6%) consisted primarily of maize husks and leaves. All quids visually identified as *Agave* were verified to be *Agave* based on phytolith analysis, while all of the non-*Agave* quids were identified as grass family based on the shapes of recovered phytoliths. One quid (which appeared to be a part of a corn husk) could be tentatively identified as maize due to the presence of phytoliths in the shape of a three-dimensional cross, which have been attributed to *Zea mays* in previous work [29].

### Phytolith content

The cellulose content in 15 of the 25 phytolith extractions of *Agave* quids was successfully digested. Between 8% and 22% of the weight of Agave quids was composed of phytoliths with an average of 15% phytolith content overall. Four extractions of grass quids were successful with an average of 8% phytolith content. However, our methods were not successful in measuring the weight of the phytoliths recovered from all of the quids. This is because chemical processing did not completely dissolve the plant fibers and there was no way to eliminate contaminant sand in ten quids.

### Tooth impression casting

**Fig 5:**
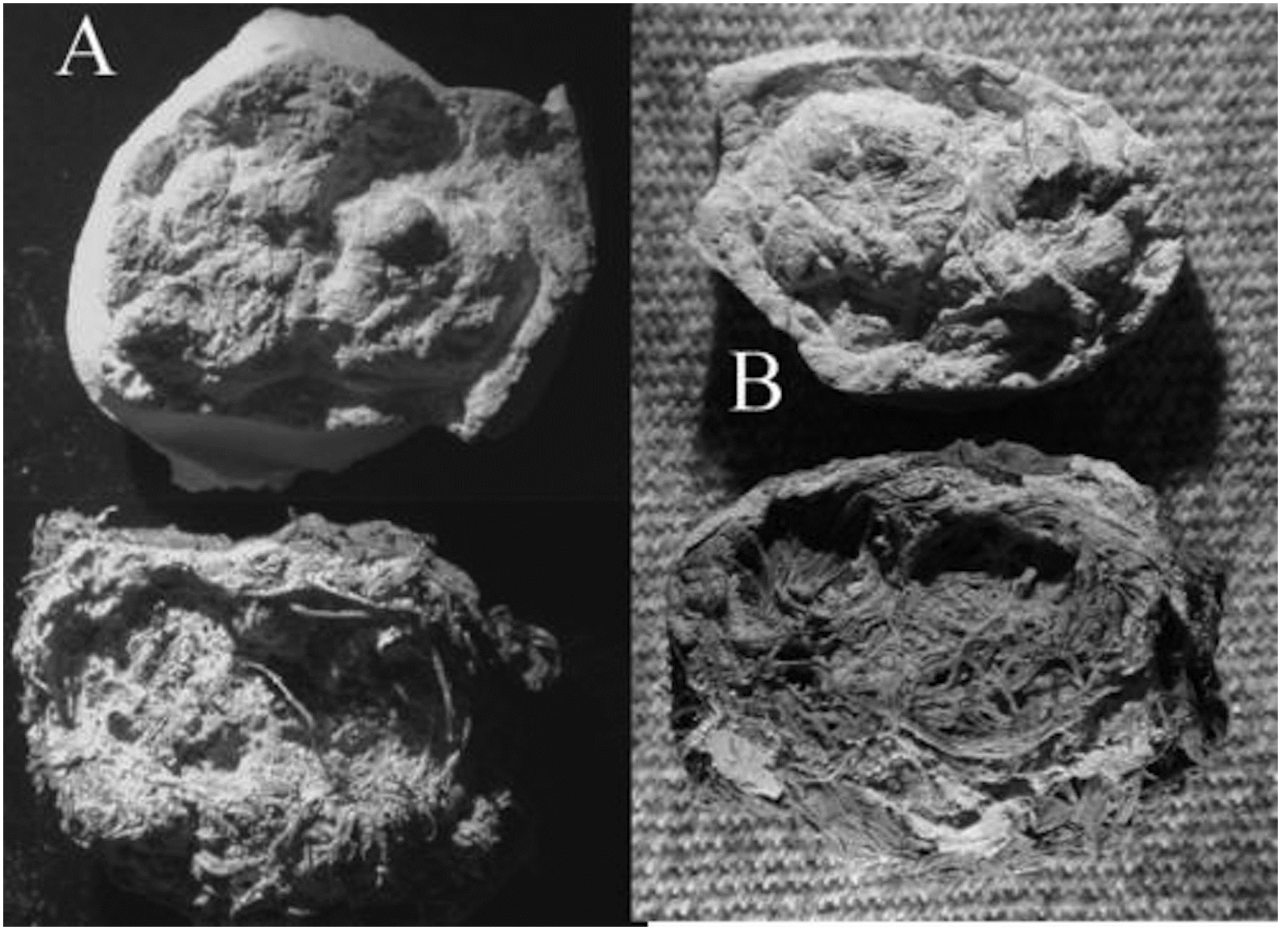
Despite the fibrous nature of quids, casts of recognizable teeth could be recovered. In the case of 5a, a cast of two molars was made but the fiber has marred the resolution of one molar. In contrast, the cast of 5b shows good resolution of both molars. This is due to a better separation of the Labstone in 5b relative to 5a. Some Labstone can be seen adhering to the 5a quid but not the 5b quid.

**Fig 6:**
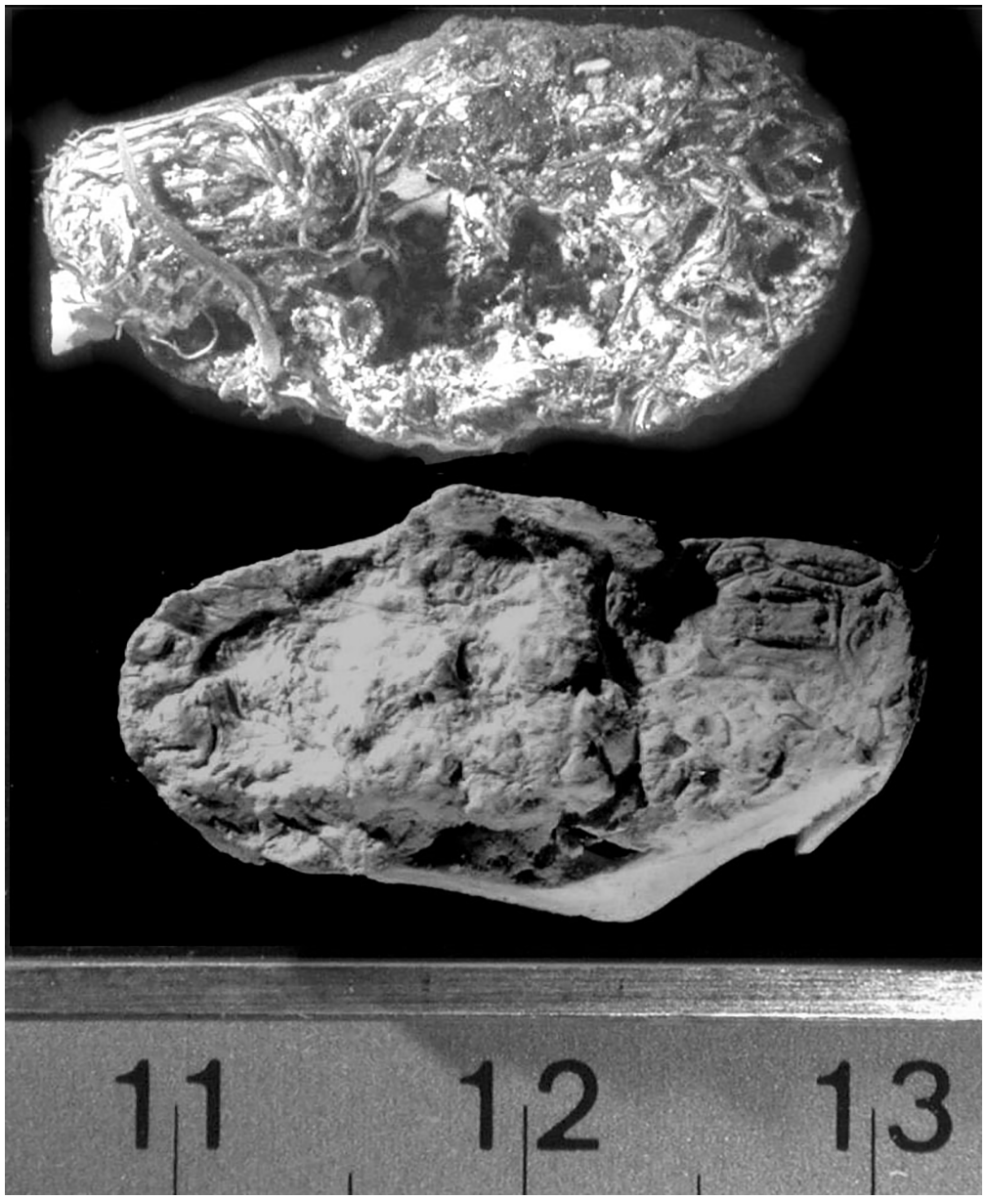
In some cases, good casts were recovered from quids that appeared to be worthless for dental analysis. In this case, the quid shows an apparent imperfect dental impression. However, the cast recovered from this quid shows a recognizable molar cusp pattern.

Casts were successfully made from 50 quids (Figs 5 and 6; Tables 1 and 2). Of these, 49 had identifiable dental features that enabled identification of individual teeth upon casting. Two casts that were made revealed part of the dental arcade but only vague outlines of teeth. The margins between enamel and dentin in casts of worn teeth were not distinct enough for application of dental wear scoring following Scott’s [30] and Smith’s [31] methods. Nearly all of the teeth that were cast were anatomically identifiable as to the type of tooth (Tables 1 and 2).

**Table 1:** Summary of observations for 15 apparent subadult individuals. Identification of subadult dentitions was made on the relative size of the dental arcade, retention of deciduous teeth, and/or evidence of unerupted molars.

N =	Teeth evident in casts made from quids	Estimated age of individual(s)
2	Deciduous central and lateral incisors, no evidence of canines	< 7 years
1	central and lateral incisors, canine, 1^st^ and 2^nd^ premolars	>12 years
1	lateral incisor, canine, 1^st^ and 2^nd^ premolars	>12 years
2	Deciduous 1^st^ and 2^nd^ molars	3–7 years
1	2^nd^ premolar, 1^st^ and 2^nd^ permanent molars, no 3^rd^ molar	12–21 years
5	1^st^ and 2^nd^ premolars, 1^st^ molar	>12 years
2	1^st^ and 2^nd^ premolars	>12 years
1	2^nd^ premolar, 1^st^ molar	>12 years

**Table 2:** Observations of teeth present in 36 adult casts. In general, there is a tendency for adults to chew quids with the posterior dentition.

N =	Teeth observed in cast from quids
1	Canine, 1^st^ and 2^nd^ premolars, 1^st^ and 2^nd^ molars
2	Canine, 1^st^ and 2^nd^ premolars, 1^st^ molar
3	1^st^ and 2^nd^ premolars, 1^st^ and 2^nd^ molars
6	1^st^ and 2^nd^ premolars, 1^st^ molar
7	2^nd^ premolar, 1^st^ and 2^nd^ molars
2	2^nd^ premolar, 1^st^ molar
3	1^st^, 2^nd^, and 3^rd^ molars
7	1^st^ and 2^nd^ molars
1	2^nd^ and 3^rd^ molars
1	Isolated molar, (partly edentulous)
1	Edentulous imprint of anterior dental arcade
2	Cast too imperfect to observe distinct teeth

**Fig 7:**
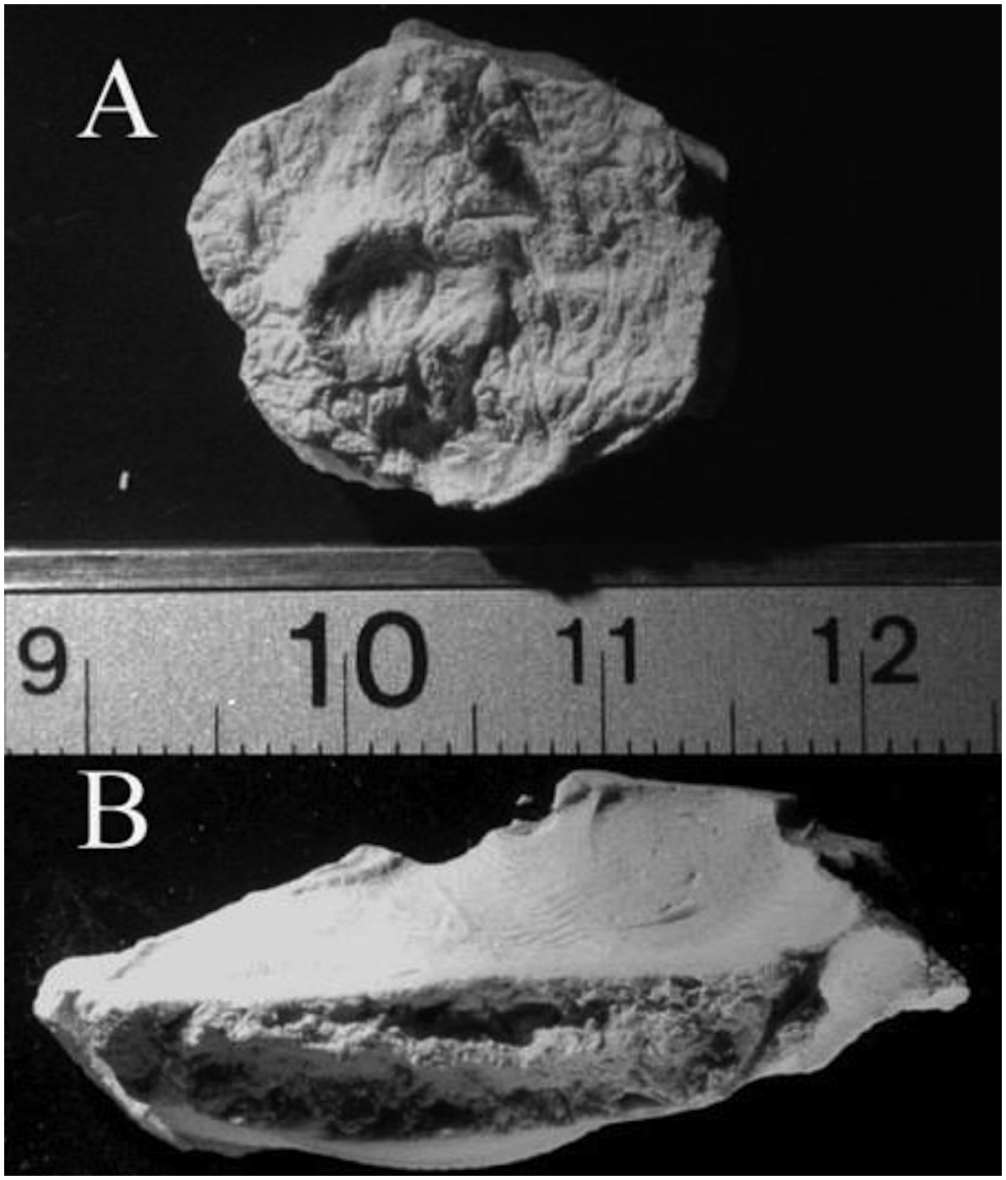
Children’s dentition was cast from some quids. Fig 7a shows a subadult molar; 7b is the cast of four incisors. Scales are in centimeters.

Dentition from young children was evident (Fig 7 and Table 1). Size and eruption patterns gave a rough estimate of the age of the chewer and quid chewing patterns associated with age were noted. In short, quids from younger people had more impressions of anterior teeth (incisors, canines) while quids from older people had more impressions of posterior teeth (premolars, molars).

### Assessment of dental wear

**Fig 8:**
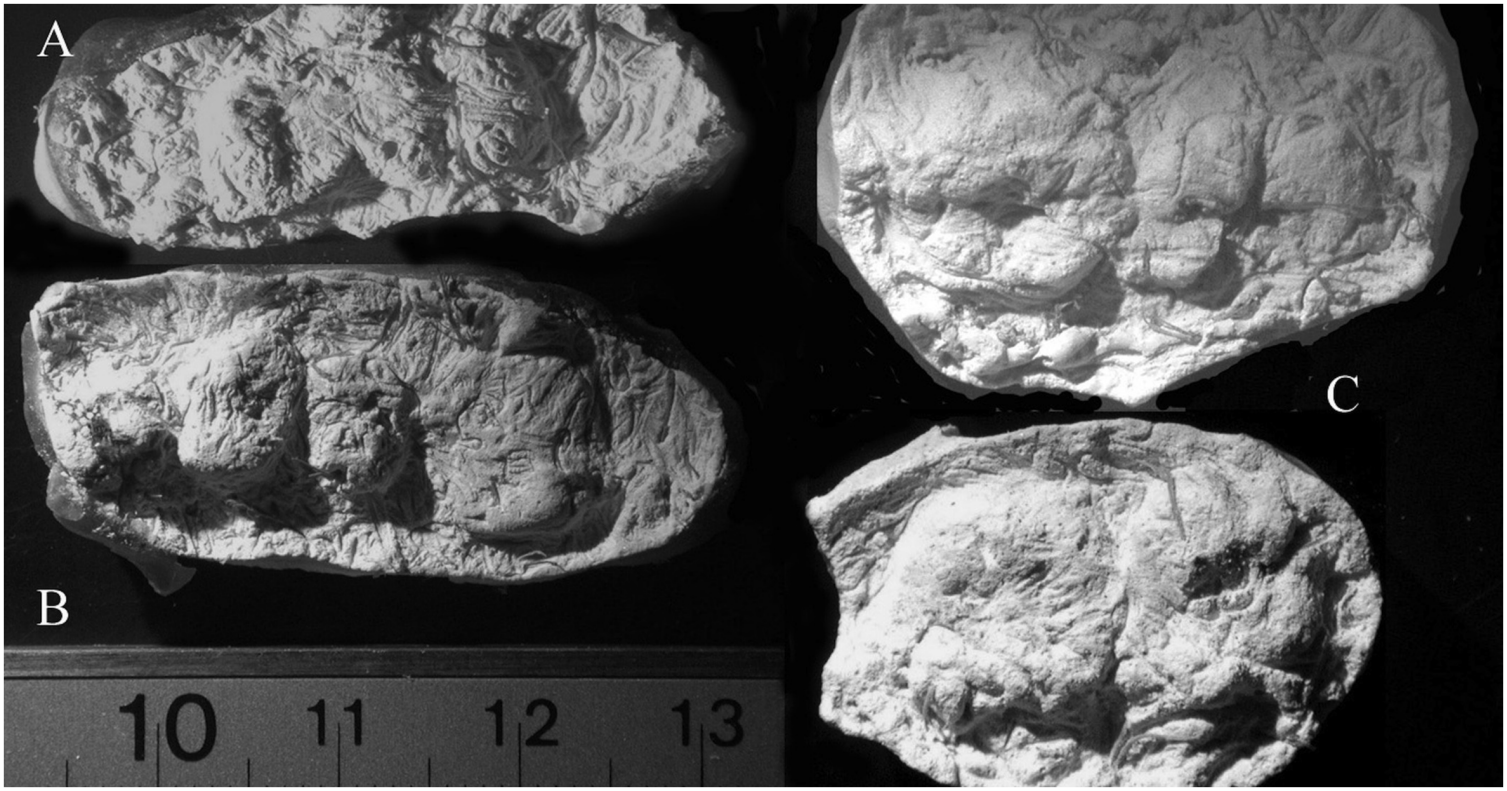
This shows the adult dental casts from three quids. Casts 8a and 8b show recognizable teeth with no apparent dental wear. 8c shows two casts made from the upper and lower sides of a single quid. This is the partial cast of the upper and lower dentition of a single individual. Scale is in centimeters and is for a and b only.

**Fig 9:**
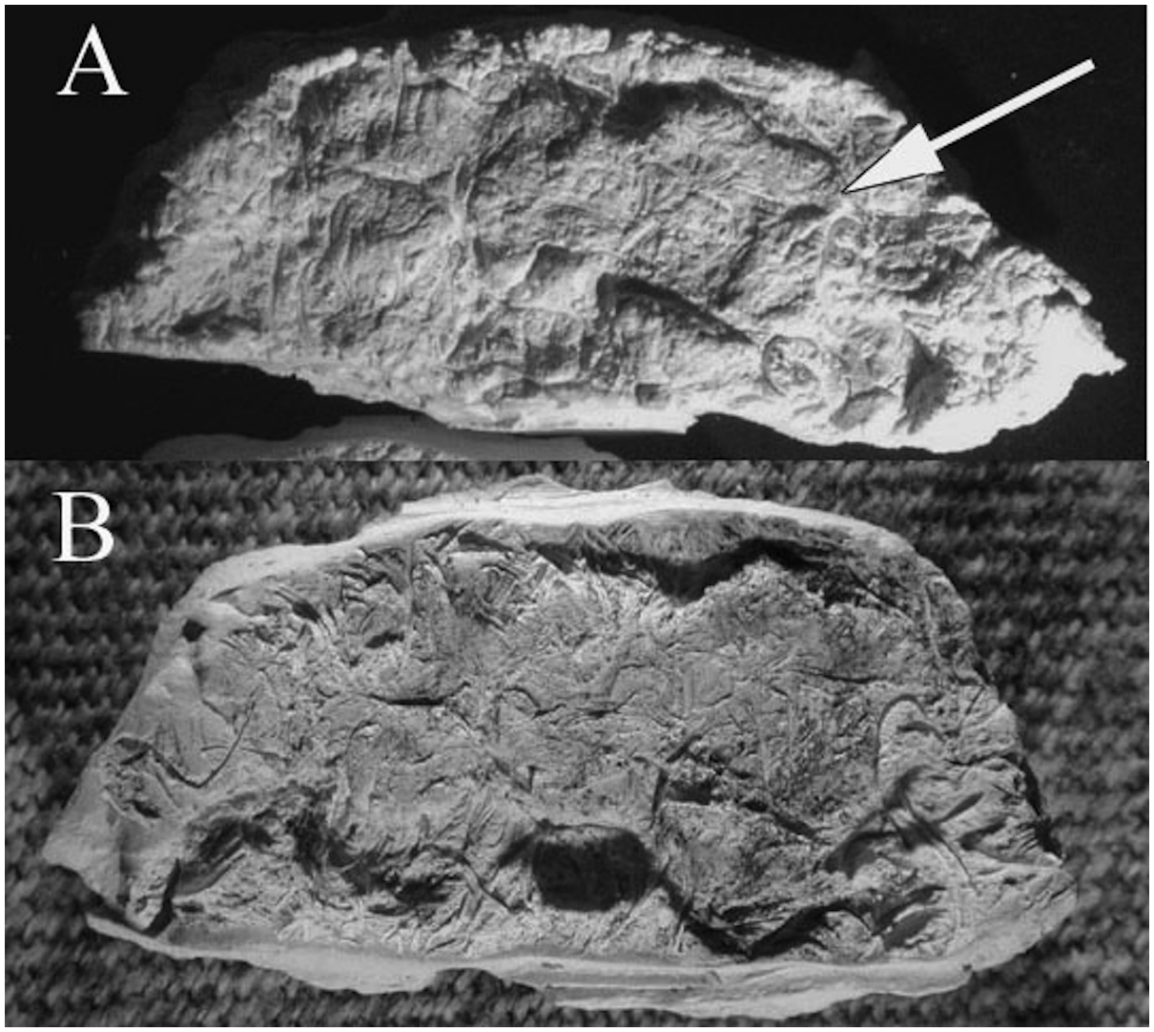
Evidence of dental wear. The arrow in 9a points to a molar cast that has three ridges around the margins of the occlusal surface. It is very likely that this represent dental wear of the softer dentin in the center of the tooth with a raised ridge of harder enamel around the margins of the surface. Fig 9b shows flat wear of molars from one quid. Cusps are not visible.

**Fig 10:**
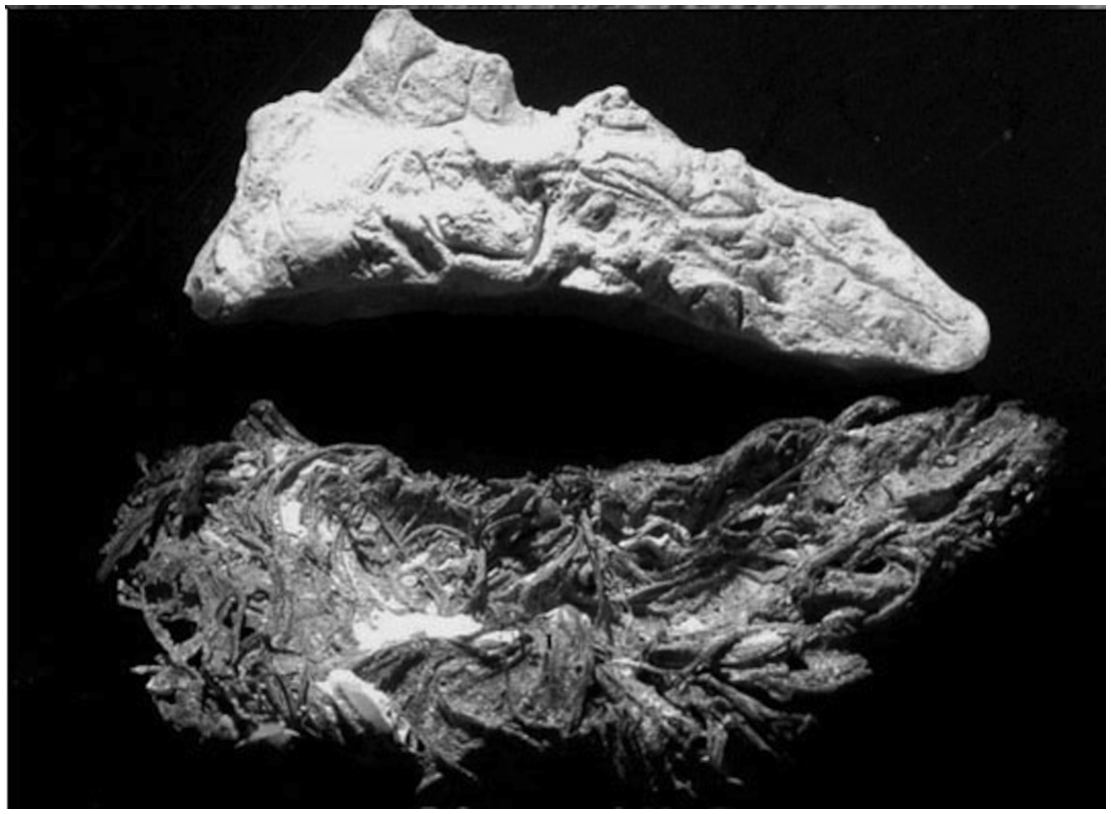
This is the cast of an anterior adult mandible or maxilla in the incisor region. No incisors are evident in this cast. Therefore, we interpret that this cast is from an individual who was at least partially edentulous. Edentulous conditions are normal after 30 years of age among populations who subsist on desert succulents (Anderson 1967; Marks et al 1988; Danielson and Reinhard 1998).

**Fig 11:**
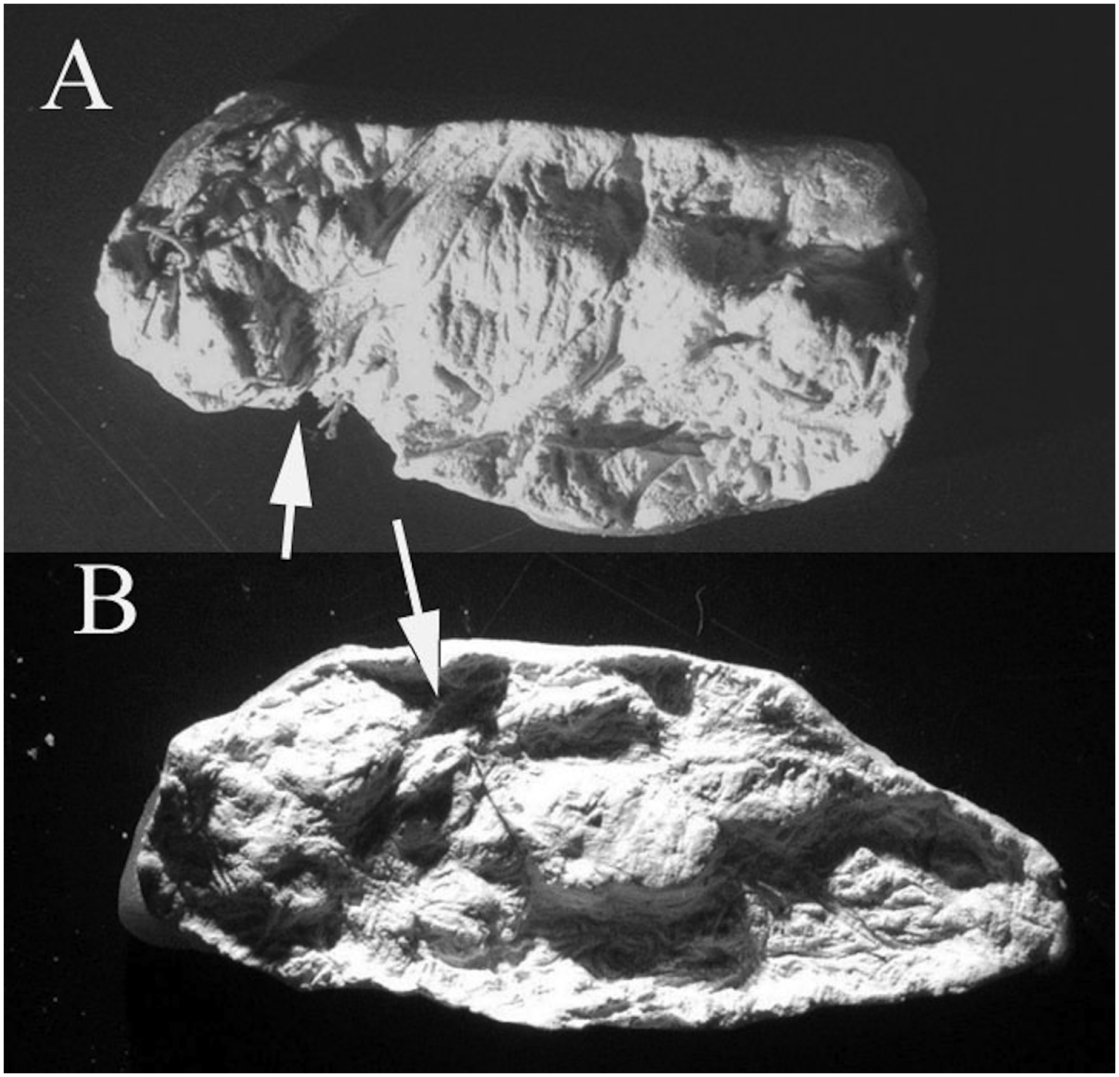
Some casts are more difficult to interpret than others. The arrow in 11a points to a molar that is apparently erupting in the cast of a subadult individual. The arrow in 11b points to an object that could be a crowded tooth or a tooth in the process of erupting.

In addition to identification of individual teeth, it was possible to observe the presence and absence of dental wear (Figs 8 and 9). The wear observed in this sample is similar to the flat wear noted for lower Pecos Archaic dentition which has previously been associated with quid chewing [5,19,20]. One cast of an anterior mandible or maxilla is of an apparent edentulous individual (Fig 10). Fig 11a shows a cast of a tooth that may be erupting in a subadult. Fig 11b shows a cast of dental crowding due to eruption. Notably, all 49 usable casts represent distinct tooth patterns.

## Discussion

All quids contained an abundance of phytoliths. On the average, 15% of the weight of *Agave* quids was composed of phytoliths. This figure. highlights the great amount of dental abrasives in *Agave* quids. In the quids, the chisel-shaped phytoliths project out of fiber bundles arranged in parallel rows in the plant tissue. This pattern resembles the surface of a kitchen grater and, like microscopic graters, a previous analysis revealed incised parallel lines on the teeth of quid chewers [5].

The question arises, were there other dietary abrasives in the diet that may have contributed to dental wear in this population? As reviewed by Reinhard and Bryant [32], grinding stone grit can be evidenced in coprolites. Sources of dental abrasives in the Rio Zape diet have previously been assessed through coprolite analysis on the La Cueva de los Muertos Chiquito collection [33]. In this study, Meade analyzed 43 coprolites from diverse proveniences and for all types of dietary remains including pollen, seeds, fibers, phytoliths and mineral grit. Traces of sand were found in 24 coprolites. However, the maize consumed at the site was unground. In contrast to other sites where extensive grinding of domestic and wild grains is evidenced [34], the coprolites from Rio Zape consist of unmilled remains. The origin of the sand was problematic. For this article, a sample of cave matrix was processed for comparative macrofossils and microfossils. Additionally, the retained, unprocessed coprolites were examined. The coprolites were perforated by coprophagous insects as evidenced by tiny holes in the coprolite surfaces. In the matrix sample, sand grains smaller than 250 micrometers were observed. We believe that the mineral grit in the processed coprolites was not a result of ingesting fragments of grit from grinding stone. Instead, it is likely that grit was introduced into the samples from post depositional, taphonomic conditions.

Biogenic crystals were abundant in the coprolites. Hundreds of phytoliths per gram of sample were recovered from the samples. The most important of these were agave, cactus and squash. *Agave* phytoliths were recovered from 25 (83%) of the coprolites. Cactus, prickly pear, phytoliths were present in 12 samples and squash phytoliths were present in 5 samples. In prevalence and abundance, *Agave* phytoliths were overwhelmingly recovered from the coprolites [33]. This evidence shows that the people living in this cave were eating a large amount of *Agave* (as is also shown by the quids), that *Agave* was the most common food eaten at the site, and that a large number of phytoliths were being extracted from the fibers during chewing. This implies that the teeth were being abraded by repeated exposure to these phytoliths as people chewed the quids.

The macroscopic and pollen evidence of foods from the coprolites include fruits from ground cherry, maize, sunflower, pigweed and goosefoot. Far more common than seeds, kernels, and achenes, course fiber composed the majority of the coprolites and accounted for an average of 60% of the average coprolite weight. *Agave* epidermis was also found. Entire, swallowed quids were evident in some of the coprolites. Thus, the macroscopic evidence from this previous study supports the phytolith evidence that *Agave* was the major food at the site and that it is the main source of dietary abrasives [33].

The casts created from these quids attest to the fact that dental wear was a problem for the inhabitants of La Cueva de los Muertos Chiquitos. Some of the casts show a complete flattening of the occlusal surface of the teeth meaning that all of the cusps had been worn away. Three quids show evidence of loss of the molars, and in one case the anterior teeth. This provides indirect evidence that the diet consumed by the inhabitants of this site was abrasive. Analysis of the quids and coprolites shows that the most consistent source of dietary abrasives was from the prehistoric dietary use of *Agave*. It is noteworthy that grit identifiable as grinding stone residue was not observed in our analysis nor that of Meade [33].

The present analysis supports Danielson and Reinhard’s hypothesis that desert succulent consumption causes dental wear in cultures beyond the lower Pecos region of Texas and Coahuila [5,9]. Importantly, the archaeological evidence indicates that the inhabitants of La Cueva de los Muertos Chiquitos had a diverse subsistence strategy that included cultivated plants such as maize and beans [26,33]. It is likely that these inhabitants cultivated *Agave* since the site is well within the range of cultivation of this plant. Therefore, *Agave*-induced dental wear was a phenomenon that was not restricted to hunter-gatherers.

*Agave* and other related plants have been life-sustaining foods for people in the deserts of Mexico and the Southwestern United States for millennia. Although intensive processing is required to make *Agave* edible, many groups felt it worthwhile nonetheless to go through the energy expenditure to consume the sweet, yet fibrous food. Unfortunately, this food appears to have its price in the form of dental abrading phytoliths. Examination of tooth impression casts from *Agave* quids found in this cave in Mexico documents this dental wear.

The use of *Agave* in northern Mesoamerica was extensive among hunter-gatherers and agriculturalists [35,36]. Previous work in Mesoamerica made a connection between desert succulent diet and dental disease. Excavations in the Tehuacan Valley of southern Mexico unearthed 10,000 *Agave* (maguey) quids and 70 skeletons from five caves. Tehuacan dentition showed marked dental attrition and tooth loss which was attributed to “the course, abrasive diet of people who gathered a large part of their food from the natural vegetation, among which maguey and various cactus are present” (page 106 in [37]). The attrition was noted in children as young as eight years old as well as adults. The analysis of quids (Table 26 in [36]) and coprolites [22] demonstrated an unbroken record of *Agave* subsistence for the Tehuacan Valley. As a result, the analysis of quids can be seen to be a great contribution to the study of these populations.

## Conclusions

Our study presents a method for evaluation of the relation between dental wear and subsistence for sites containing quids. By casting dentition from the quids and analyzing the abundance and nature of phytoliths in quids, one can see the origin of dental abrasives in plant foods and the result of wear in the casts. As a result, we encourage archaeologists, paleoethnobotanists, and biological anthropologists to collect and analyze quids from sites as standard practice.
